# A CTSA One Health Alliance (COHA) survey of clinical trial infrastructure in North American veterinary institutions

**DOI:** 10.1186/s12917-021-02795-z

**Published:** 2021-02-25

**Authors:** Sarah A. Moore, Angela McCleary-Wheeler, Joan R Coates, Natasha Olby, Cheryl London

**Affiliations:** 1 Comparative and Translational Medicine Program, The Ohio State University College of Veterinary Medicine, Columbus, USA; 2 Columbia College of Veterinary Medicine, University of Missouri, Columbia, USA; 3 North Carolina State University College of Veterinary Medicine, Raleigh, USA; 4 Cummings School of Veterinary Medicine, Tufts University, Medford, USA

## Abstract

While a necessary step toward enhancing rigor and reproducibility of veterinary clinical trials conducted on the translational spectrum includes understanding the current state of the field, no broad assessment of existing veterinary clinical trial resources has been previously conducted. Funded by a CTSA One Health Alliance (COHA) pilot award, the goal of this project was to conduct an electronic survey of North American Veterinary Colleges regarding practices in veterinary clinical trial review, approval, conduct, and support in order to identify opportunities to leverage existing resources and develop new ones to enhance the impact of veterinary and translational health research.

A total of 30 institutions were invited to participate in the survey and the survey response rate was 73 %. The most common source of funding noted for veterinary clinical research was industry (33 %); however, respondents reported that only 5 % (3.7–11.0) of studies were FDA-regulated. Respondents indicated that most studies (80 %); conducted at their institution were single site studies. Study review and approval involved the IACUC either solely, or in combination with a hospital review board, at 95.5 % of institutions. Workforce training related to clinical research best practices was variable across institutions.

Opportunities were identified to strengthen infrastructure through harmonization of clinical research review and approval practices. This might naturally lead to expansion of multi-site studies. Based on respondent feedback, future workforce development initiatives might center on training in the specifics of conducting FDA-sponsored research, Good Clinical Practice (GCP), clinical study budget design, grants management, adverse event reporting, study monitoring and use of electronic data capture platforms.

## Background

Well-executed veterinary clinical research provides protections for veterinary patient participants, their owners, and investigators while ensuring that data obtained provide maximal benefit from a veterinary and translational medicine perspective^1,2^. Understanding of the current infrastructure associated with veterinary clinical research is essential for benchmarking and identifying where to strategically focus need-based initiatives to grow and improve the clinical research landscape. The CTSA One Health Alliance (COHA) is a group of academic veterinary medical centers partnered with local schools or colleges of medicine on translational science initiatives through NIH-supported Clinical and Translational Sciences Awards (CTSA). A major goal of COHA is to leverage natural animal models of disease, by way of veterinary clinical trials, to promote advances in health care that can benefit both animals and people by promoting improved understanding of pathophysiology and treatment of conditions shared across species^3^. While understanding the current state of the field is a necessary step toward enhancing rigor and reproducibility of veterinary clinical trials conducted on the translational spectrum, no broad assessment of existing veterinary clinical trial resources has been conducted previously. Funded by a COHA pilot award, the goal of this project was to conduct a survey of North American veterinary colleges to document current practices in veterinary clinical study review, approval, conduct, and support in order to identify opportunities to leverage existing resources and develop new ones that can enhance the impact of veterinary and translational health research.

## Methods

### Survey development process

Funded by the CTSA One Health Alliance (COHA) pilot program, an electronic survey was developed to collect information on institutional demographics, methods of clinical research oversight, use of risk-based assessment and monitoring, and details on quality assurance and other monitoring practices. The finalized version of the survey was formatted using Qualtrics (www.qualtrics.com), a software program for web-based survey administration. The survey consisted of 49 questions with structured response options provided via a check box method or free-text short answer responses. For some questions, an “other” option allowed respondents to provide additional comments.

 Informed consent was obtained from all respondents at the beginning of the survey. Because the survey asked only for data associated with institutional processes, and no personal data or opinions were solicited, it was determined that review and approval by the University Institutional Review Board (IRB) was not required. On November 4, 2019 the survey was distributed electronically to all continental North American veterinary schools per a list provided by the American Association of Veterinary Medical Colleges (AAVMC). The survey remained open for 6 weeks. Two electronic reminders were sent to encourage survey completion. One was sent half way through the survey window and one was sent two days prior to close of the survey.

### Distribution

The survey targeted veterinary schools/colleges and academic veterinary medical hospitals across the United States and Canada. Electronic survey distribution occurred via email with a personalized link for survey access sent directly to each university’s associate dean of research with carbon copies sent to university veterinary medical hospital directors, and clinical trial directors (where applicable). An accompanying email explained the purpose of the survey and asked recipients to provide a single response representing their institution. For the purposes of this survey, respondents were instructed that veterinary clinical research was defined as “any prospective research involving client-owned animals” and the terms “veterinary clinical research” and “veterinary clinical trial” could be considered interchangeable. Recipients were also provided a word document listing the survey questions to allow easy review and discussion, and were encouraged to collaborate with or forward the survey to whoever had the most detailed knowledge of their institution’s veterinary clinical research portfolio.

### Statistical analysis

After closure of the survey, data was analyzed and descriptive statistics were computed using Qualtrics software. Categorical data are reported as total percentages of respondents that selected the category, and continuous data are reported as median and 90 % confidence interval.

## Results

### Overall response and respondent demographics

A total of 30 institutions were invited to participate in the survey. Complete responses were received from 22 institutions, constituting an overall survey response rate of 73 %. No partial survey responses were received. All respondents indicated that their college/school, department, or hospital conducts veterinary clinical research as part of its routine business. The role of the individual completing the survey was recorded as Associate Dean of Research or equivalent in 36.4 % (*n* = 8) of cases, a faculty member (18.2 %; *n* = 4), a staff member (18.2 %; *n* = 4), Director of Clinical Trials Office or equivalent (13.6 %; *n* = 3), Dean of the Veterinary College/School (4.5 %; n = 1) or Other (9.1 %; *n* = 2). In 68.2 % (*n* = 15) of instances, institutions indicated they were COHA members, while 31.8 % (*n* = 7) were not. Of those institutions that were not currently COHA members, 4 indicated they were actively working toward CTSA funding through partnership with a local school or college of medicine.

### Clinical research infrastructure

When asked whether their institution currently had a veterinary clinical trials office or other centralized veterinary clinical research unit, 59.1 % of respondents indicated yes and 40.9 % indicated they did not. For those institutions with a veterinary clinical trials office, the median total personnel full time equivalent (FTE) associated with the office was 4.3 (2.2-6.0). Of these, the median FTE within the office devoted to patient care was 3.5 (1.5–4.4) and the median FTE devoted to administrative/managerial duties was 1.0 (0.5–1.6).

### Clinical research funding

**Fig. 1:**
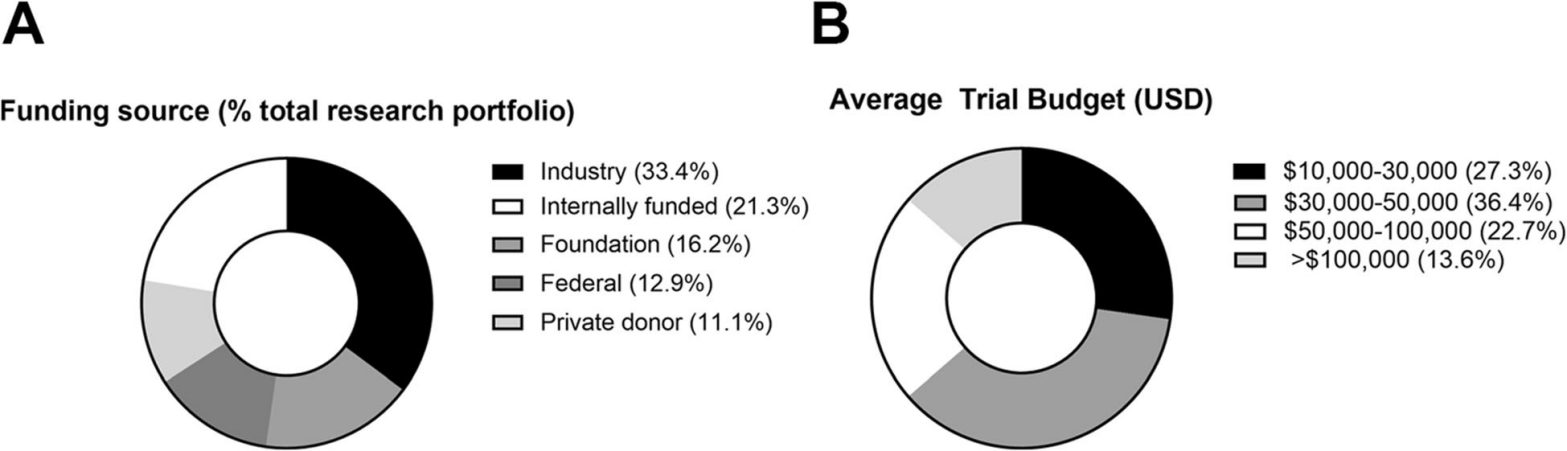
Funding sources (**a**) and average total budgets (**b**) for veterinary clinical trials conducted across the continental United States and Canada

Funding sources, as a percentage of an institution’s clinical research portfolio, and average total clinical study budgets for each respondent institution are detailed in Fig. 1. The most common source of funding noted for veterinary clinical research was industry (33.4 %; 23.2–43.5). Additional sources included studies funded by principal investigator “start-up” or other internal funds (21.3 % ;14.0-28.6), external funding by foundations (16.2 %; 11.1–21.5), federal grants (12.9 %; 7.4–18.5), and gifts/charities/private donations (11.1 %; 4.6–17.7). Despite the high number of industry-sponsored trials, respondents reported that only 5 % (3.7–11.0) of total trials were FDA-regulated. With respect to salary recovery, respondent institutions indicated that 50 % (33.3–65.0) of their trials provided some level of salary support for all or part of the study team. The most commonly selected average total study budget (USD) was $30,000–50,000 (36.4 % response rate).

### Clinical study design and conduct

**Fig. 2:**
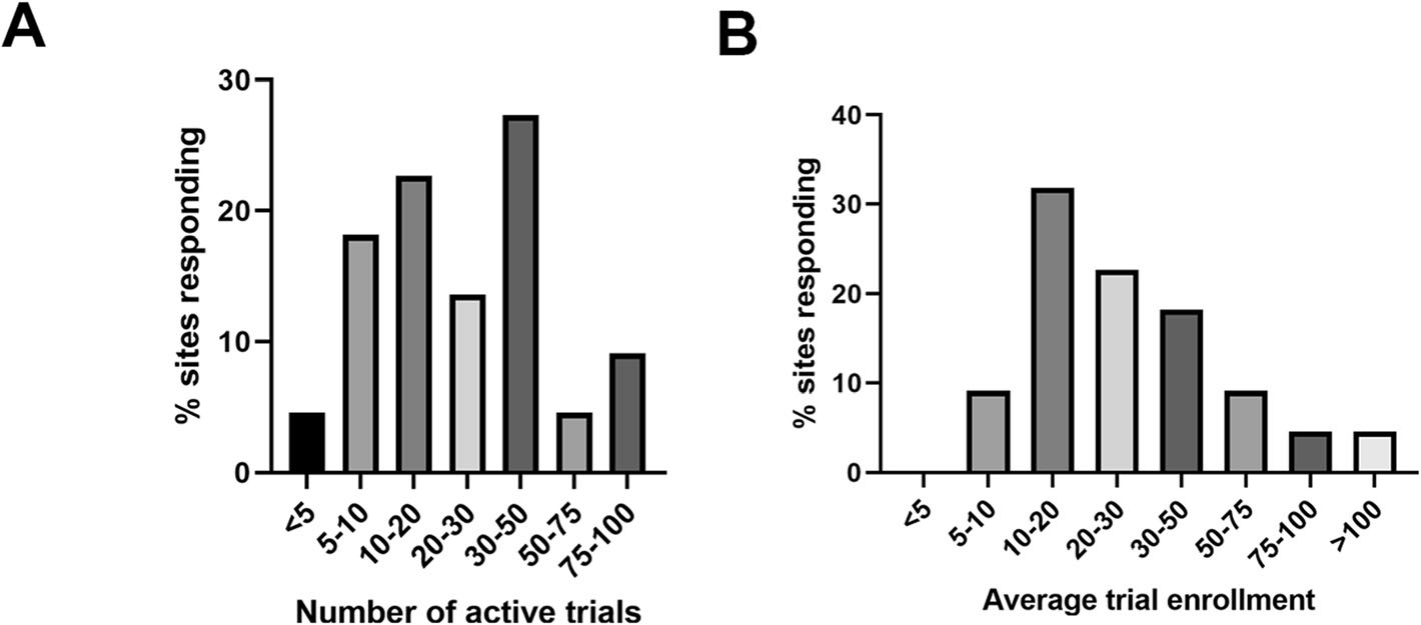
Veterinary clinical trial demographics, including average number of animals enrolled per study (**a**) and average number of currently active clinical studies per institution (**a**) across North America

Institutions were asked to indicate the percentage of their clinical research portfolio that met an assortment of criteria related to study design and conduct. Figure 2 details responses associated with average number of active trials and average per trial patient enrollment over the last three years across respondent institutions. When asked to estimate how many trials were currently active at their institution, the most commonly selected option was 30–50 active trials (27.3 % response rate) while a small number of responding institutions indicated less than 5 active trials (4.5 %) or 75–100 active trials (4.5 %). The most commonly selected average total patient enrollment per trial over the last 3 years was 10–20 (31.8 % response rate) or 20–30 (22.7 % response rate).

When respondents were asked whether their institution had conducted veterinary clinical trials incorporating the use of recombinant or synthetic nucleic acid molecules (e.g. gene therapy, recombinant DNA, vaccine studies) or cell based therapies over the last 5 years, a majority (63.6 %) indicated having participated in studies evaluating recombinant or synthetic nucleic acids in client owned animals. With respect to conducting studies incorporating cell-based therapies, 77.3 % of institutions indicated having done so.

### Multi‐institutional study participation

With respect to multi-institutional study participation, respondents indicated that most studies (80 %; 65.5–84.4) conducted at their institution were single site studies while only 5 % (4.6–13.0) were multi-site studies where the respondent was the lead institution. When asked whether they had served as the lead site or subcontract for studies involving private practice facilities, respondents indicated “yes” in 77.3 % and 22.7 % of instances, respectively. A smaller number (22.7 %) of institutions indicated having served as the lead site on a clinical study where secondary sites were located outside of the United States. Additionally, 18.2 % indicated having served as a secondary study site where the primary site was located outside of the United States.

### Study review, approval, and monitoring

Institutions were asked to supply information regarding how veterinary clinical research studies were reviewed and approved prior to initiation at their institution. 50 % indicated that they were reviewed only by an institutional animal care and use committee (IACUC), 4.5 % indicated that they were reviewed only by a hospital-specific veterinary review board, and 45.5 % indicated that they were reviewed by both an IACUC and a hospital-specific veterinary review board. This review included evaluation of study consent forms at 95.5 % of institutions, and most (90.1 %) provided a consent template to guide investigators with development of the form. Once initiated, veterinary clinical studies underwent some form of routine post-approval monitoring at 59.1 % of respondent institutions. Post-approval monitoring occurred yearly (69.2 %), every three years (23.1 %), or other (7.7 %) where further information regarding other frequencies was not provided. The process included the following: a request for the PI to provide the number of animals enrolled to date (32.4 %); a request for the PI to update the study protocol as necessary (27.0 %); a request for the PI to update the study team as necessary (21.6 %); an audit of consent forms (8.1 %); an audit of research records (5.4 %); and other (5.4 %). Respondents who selected “other” were provided a free-text entry option to allow them to elaborate on what “other” items were monitored or reviewed. No additional information was provided by any of the respondents.

With respect to adverse event monitoring and reporting, 50 % of respondents indicated that their IACUC or hospital review board had a formal process for adverse event reporting in veterinary clinical studies. Of those institutions with formal reporting mechanisms, 63.6 % indicated that adverse events were reported to the IACUC, 18.2 % indicated that they were reported to their hospital’s review board, and 18.2 % indicated that they were reported only to the study sponsor.

 Institutional respondents were asked a series of questions regarding how heavily certain elements of ethical study design were weighted for consideration during the study review and approval process. Detailed responses are reported in Table 1. All institutions indicated most elements presented in the survey were considered at least moderately important during the review and approval process; however, items considered extremely important by the majority of respondent institutions included informed consent and respect for potential and enrolled subjects, while items such as social value, scientific validity, and favorable risk/benefit ratio were weighted less heavily.

**Table 1:** Detailed responses regarding weighted consideration of various aspects of research conduct during the study review and approval process. Respondents were asked to quantify how heavily various factors were considered during study review and approval. Number indicates percentage of respondents indicating each level of importance

Factor	Extremely important	Very important	Moderately important	Slightly important
**Favorable risk/benefit ratio**	36.4	36.4	27.3	0
**Informed consent**	81.8	13.6	4.5	0
**Respect for potential and enrolled subjects**	72.7	22.7	4.5	0
**Scientific validity**	40.9	27.3	18.2	13.6
**Social and clinical value**	22.7	36.4	31.8	9.1

### Research training, conduct, and best practices

Respondents were asked a series of questions focusing on research best practices and patterns at their institution. Detailed responses are reported in Table 2. When asked to estimate how frequently investigators conducted a feasibility analysis prior to initiation of a clinical trial, the most common answer was “sometimes” (45.5 %), with “always” or “most of the time” selected by 18.2 % and 27.3 % of institutions, respectively. When asked about the frequency of use of electronic laboratory notebooks or electronic study records for clinical research data capture, the most common response was “sometimes” (54.6 % of institutions) with “always” or “most of the time” selected by 9.1 % and 18.2 % of respondents institutions, respectively. When asked about the frequency with which investigators will have completed training in good clinical practice (GCP), the most common response was “sometimes”, selected by 63.6 % of institutions. Only 13.6 % of institutions indicated GCP training was completed “always” or “most of the time” by their investigators prior to initiating clinical research at their facility. Completion of formal training in responsible conduct of research (RCR) was indicated as “always” or “most of the time” complete by investigators prior to initiating clinical research at 63.6 % of institutions. Formal training in budget development was undertaken by investigators “always” or “most of the time” at 4.6 % of institutions, and no institution indicated that formal training in grants management was completed “always” or “most of the time” by investigators prior to initiating clinical research. When asked about the use of a data safety monitoring committee or other entity for monitoring patient safety and treatment efficacy while a study is on-going, only 18.2 % of institutions indicated that this occurred “always” or “most of the time”.

**Table 2:** Frequency of conduct of various “best practice” activities across veterinary institutions in North America. Respondents were asked to quantify what percent of the time an investigator at their institution would perform a specific task with respect to study design or conduct. Number indicates percentage of respondents selecting each frequency

	Frequency (%)				
**Task**	**Always**	**Most of the time**	**About half the time**	**Sometimes**	**Never**
**Conduct a feasibility analysis of any type**	18.1	27.3	9.1	45.5	0
Consider disease frequency in the general veterinary population	36.4	54.6	0	9.1	0
Consider disease frequency Hospital or program-specific caseload	45.5	45.5	4.5	4.5	0
Consider existing treatment patterns or guidelines	36.4	45.5	0	18.2	0
Consider availability of alternative drugs or treatments	18.2	54.5	0	27.3	0
Consider presence of competing trials within the hospital	23.8	33.3	9.5	28.6	4.8
Consider presence of competing trials within the geographical region	0	23.7	4.6	59.1	13.6
**Complete a sample size determination prior to initiating a study**	27.3	40.9	9.1	18.2	4.6
**Use an electronic laboratory note book or study record for clinical data capture**	9.1	18.2	13.6	54.6	4.6
**Complete a formal GCP training program prior to initiation of their first clinical trial**	13.6	0	4.6	63.6	18.2
**Complete a formal RCR training program prior to initiation of their first clinical trial**	36.4	27.3	9.1	27.3	0
**Complete formal budget training program prior to initiation of their first clinical trial**	0	4.6	4.6	45.5	45.5
**Complete a formal grants management training program prior to initiation of their first clinical trial**	0	0	4.6	54.6	41.0
**Read and/or sign a PI agreement dealing with the Institution’s policies procedures and expectations for investigators participating in veterinary clinical research**	9.1	9.1	4.6	40.9	36.4
**Utilize a data safety monitoring committee or similar entity**	13.6	4.6	0	54.6	27.3

*GCP* Good Clinical Practice. *RCR* responsible conduct of research. *PI* principal investigator

Several questions focused on quantitative and logistical considerations surrounding feasibility of clinical research conduct. Institutions indicated that investigators would have completed a sample size determination prior to study initiation “always” or “most of the time” in 68.1 % of cases. When asked about the *a priori* consideration of factors that might influence study accrual, respondents indicated that researchers considered the following criteria “always” or “most of the time”: Disease frequency in the general population (91.0 %); hospital or program-specific caseload (91.0 %); existing treatment patterns or guidelines (81.8 %); availability of alternative drugs or treatments (72.7 %); presence of competing trials within the hospital (57.1 %); and presence of competing trials in the geographical region (22.7 %).

### Publication of study designs and dissemination of results

The survey asked several questions regarding institutional familiarity with and use of the AVMA Animal Health Studies Database (AAHSD), which serves as a centralized platform for publication of study designs for newly initiated and ongoing veterinary clinical research, and has a goal of assisting with dissemination of study conduct and results to both veterinarians and lay persons across North America. Survey respondents indicated that they “were” or “were not” aware of the existence of the AAHSD as a veterinary clinical research marketing tool in 81.9 and 18.2 % of cases, respectively. Of respondents who were aware of the database, 22.7 % indicated that their institution did not post any trials to the AAHSD. Of those institutions who were aware of and posted to the AAHSD, respondents indicated posting approximately 42.1 % (25.4–58.7) of their active trials. Most institutions (54.5 %) indicated having a designated institutional employee who was responsible for posting trials. When asked how frequently respondent institutions followed up on their posted studies by providing eventual results or conclusions within the AAHSD, 63.6 % of those institutions who initially posted their studies the AAHSD indicated they never posted study outcomes.

## Discussion

The results of this study constitute a broad survey of veterinary clinical research practices across North America, and indicate that clinical studies are conducted at most, if not all academic veterinary institutions. Additionally, there exists universal institutional infrastructure for the review and approval of trials, whether that occurs through an IACUC which also reviews and approves research using purpose-bred animals, or through a hospital-specific review board, or both. For 95.5 % of respondent institutions, this review involved the IACUC at some level and for almost half of institutions, a separate hospital review board also reviewed studies prior to initiation. Proper review of veterinary clinical research can be complex as it requires regulatory knowledge associated with the portions of the Animal Welfare Act that might be relevant to certain projects based on scope and funding source, but also encompasses required knowledge of ethical and study design considerations (consent, risk/benefit analysis, conflict of interest, selection of relevant outcomes) that more closely resemble those reviewed by IRBs for human subjects studies. What constitutes the right body for review and approval of veterinary clinical studies is likely institution-dependent based on local expertise and available infrastructure. The American Veterinary Medical Association (AVMA) has previously provided some guidance on the appropriate constitution of review bodies for veterinary clinical research and various groups have weighed in on approaches to quality assurance^4–7^. Regardless of which body (or bodies) review and approve veterinary clinical research at a given institution, our survey results suggest that review criteria and weighting of individual importance of particular review items, varies substantially between institutions. Developing a more uniform approach to clinical study review and approval, as well as specific guidelines and training materials for both IACUC and veterinary hospital board reviewers could be helpful in harmonizing workflow across institutions and in facilitating a more harmonized approach to review of multi-center veterinary clinical studies.

A notable result of this survey was the relatively high frequency of industry-sponsored research but low frequency of FDA-regulated studies. While the survey was not designed to evaluate reasons for this, and did not capture the specific nature of these studies, the experience of the authors’ suggests that many of these studies might be pre-clinical work evaluating investigational new therapies that could later intend to seek FDA on either the human or the veterinary side approval. If so, failure to engage the FDA early on in these studies could represent a lost opportunity to allow data from client-owned dogs to serve as supplementary material for future investigational new drug (IND) process applications. Limited interaction between veterinary clinical investigators and the FDA may stem from limited investigator knowledge related to FDA processes and may present a training opportunity that could serve to enhance the use of veterinary disease models, by way of veterinary clinical trials, in the translational therapeutic development process.

The predominance of studies reported in this survey were single-site veterinary clinical studies, with respondents indicating that only 5 % of their clinical research portfolio consisted of multi-site studies. While these results are not surprising, they do reinforce the fact that veterinary researchers tend to be fairly “siloed” in their clinical research efforts. This is further reinforced by respondents who indicated limited consideration for the presence of competing trials in their geographic region. The importance of multi-center trials has been previously well-demonstrated for human clinical studies, where inclusion of multiple sites enhances generalizability of findings, ensures fair geographic representation, and greatly enhances efficiency of trial enrollment and completion. While veterinary medicine has seen some notable recent multi-center clinical research efforts by groups such as the Comparative Oncology Trials Consortium (COTC), the Canine Spinal Cord Injury Consortium (CANSORT-SCI), and the like, predominance of single-site studies may be due at least in part to logistical difficulties associated with review, approval, and conduct of multi-site trials^8–11^. Presently, veterinary clinical research review and approval in multicenter studies is managed on a site-by-site basis by local institutional committees. For multi-site studies, the current status quo of multiple local reviews creates inconsistencies in how studies are evaluated, and leads to substantial inefficiency in the rate at which multi-institutional studies reach full functional capacity across sites^6,11^. These barriers mirror those present on the human side prior to the development of the Streamlined, Multisite, Accelerated Resources for Trials (SMART) IRB, a platform designed to ease common challenges associated with initiating multi-site clinical research^12,13^. To address the need for robust, consistent, and efficient clinical study review and initiation in veterinary medicine, new initiatives and cross-institutional processes will likely be needed.

Questions in the current survey centered on research best practices identified gaps in workforce training and opportunities to develop resources addressing these. Specifically, workforce development and associated resources centered on training in Good Clinical Practice (GCP), clinical study budget design, grants management, adverse event reporting, study monitoring and use of electronic data capture platforms such as Research Electronic Data Capture (REDCap) appeared to be needed. Recently, COHA has developed and published a web-based GCP training module specific to veterinary clinical research, as well as a standardized template for adverse event reporting. Both can be accessed at https://www.ctsaonehealthalliance.org/. Neither were publically available at the time this survey was conducted.

## Conclusions

This survey demonstrated important opportunities to strengthen veterinary clinical research infrastructure through targeted efforts related to harmonization of clinical research review and approval practices which might naturally lead to expansion of multi-site studies. Additional opportunities for workforce development were identified in relationship to training in the specifics of conduction FDA-sponsored research, GCP, clinical study budget design, grants management, adverse event reporting, study monitoring and use of electronic data capture platforms. Research initiatives focusing on enhancing the veterinary clinical research landscape, with a goal of improving rigor and reproducibility, should focus on these documented areas of need.

## Data Availability

Data sharing is not applicable to this article as no datasets were generated or analyzed during the current study.
